# Supplementary material for: Enhancing Dissolution Rate and Antibacterial Efficiency of Azithromycin through Drug-Drug Cocrystals with Paracetamol
Source: Antibiotics (Basel). 2021 Aug 4;10(8):939. doi: 10.3390/antibiotics10080939 (PMC8388915; doi:10.3390/antibiotics10080939)
Supplement: Supplementary file 1 [file antibiotics-10-00939-s001.zip › antibiotics-1228553-supplementary.pdf]

antibiotics

MDPI

Article

Enhancing dissolution rate and antibacterial efficiency of Azithromycin through drug-drug cocrystals with paracetamol

Noor Ul Islam, Ezzat Khan, Muhammad Naveed Umar, Attaullah Shah, Muhammad Zahoor, Salah M. El-Bahy and Gaber El-Saber Batiha

Binary phase diagram

Physical mixtures of different molar ratios were grinded with the help of pestle and mortar for 15 minutes. Small amount of each physical mixture was taken in a capillary tube, which was subjected to thermal apparatus (Bibby Scientific Limited Stone, Staffordshire, ST15 OSA, UK) to determine their melting points. To predict the suitable mole ratio for co-crystallization preparation, binary phase were develop based on the obtained results [1,2].

Melting point data of samples of physical mixtures with different mole ratio was plotted against melting temperature, as shown in Figure S1. The formation of W-type binary phase diagram of the physical mixture (AZT-PCM, 1:1) and the corresponding melting point agrees with the proposed material, the middle of the curve indicates that cocrystal formation between AZT and PCM in 1:1 molar ration is possible [1-4]. Moreover, the melting points of cocrystal components and the resulted cocrystal material are given in Table S1. The melting point of the heterosynthons falls between both the starting material which provided enough insights regarding the successful formation of the cocrystal.

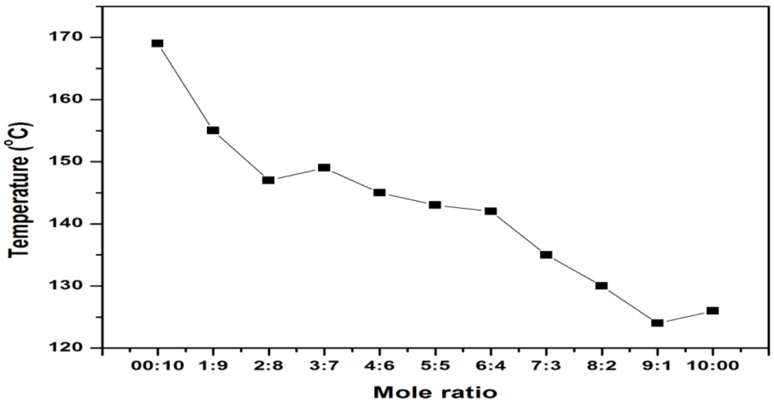

Figure S1. Binary phase diagram for AZT and PCM in different stoichiometric ratios

antibiotics

MDPI

Table S1: Melting point of raw materials and cocrystal

**sample**	**Melting point (°C)**
Azithromycin dihydrate	125-126
paracetamol	169-171
cocrystal	140-144

Micrometric studies

Flow properties of material AZT and the resulted cocrystal were determined with the intent to confirm the formation of the cocrystal or otherwise. These properties are in the opinion that the final product has completely different properties in comparison with the starting synthons, thus supporting the formation of the cocrystal.

Bulk density (BD)

Known amount of AZT and cocrystal were subjected in a measuring volume cylinder. The bulk density of the drugs and cocrystal was determined using the following formula:

$$\mathrm{BD} = \frac{\text{mass of the sample}}{\text{bulk volume of the sample}}$$

Tapped density (TD)

After measuring bulk volume, the cylinder was tapped 100 times from height of 2 cm and volume of AZT and cocrystal were calculated using the following formula:

$$\mathrm{TD} = \frac{\text{mass of the sample}}{\text{tapped volume of the sample}}$$

Carr's index (compressibility index)

Compressibility parameter of AZT and cocrystal were calculated as below:

$$\text{Compressibility index (CI)} = \frac{\mathrm{TD} - \mathrm{BD}}{\mathrm{TD}} \times 100$$

Hausner's Ratio (HR)

The Hausner's ratio for the cocrystals was calculated using the formula:

$$\mathrm{HR} = \mathrm{TD}/\mathrm{BD}$$

antibiotics

Table S2: Flow properties of AZT

Flow property	AZT	cocrystal
**Bulk volume (mL)**	1.4	1.3
**Bulk density (g/mL)**	0.71	0.769
**Tapped volume (mL)**	1.2	1.1
**Tapped density (g/mL)**	0.833	0.909
**Compressibility index**	14.76	15.4
**Hausner's ratio**	1.17	1.18

References

1. Nugrahani, I.; Utami, D.; Nugraha, Y. P.; Uekusa, H.; Hasianna, R.; Darusman, A. A. Cocrystal construction between the ethyl ester with parent drug of diclofenac: structural, stability, and anti-inflammatory study. *Heliyon* **2019**, *5*, e02946.

2. Evora, A. O.; Castro, R. A.; Maria, T. M.; Silva, M. R.; Canotilho, J.; Eusébio, M. E. S. Lamotrigine: design and synthesis of new multicomponent solid forms. *European Journal of Pharmaceutical Sciences* **2019**, *129*, 148-162.

3. Yamashita, H.; Hirakura, Y.; Yuda, M.; Teramura, T.; Terada, K. Detection of cocrystal formation based on binary phase diagrams using thermal analysis. *Pharmaceutical research* **2013**, *30*, 70-80.

4. Nugrahani, I.; Tjengal, B.; Gusdinar, T.; Horikawa, A.; Uekusa, H. A Comprehensive Study of a New 1.75 Hydrate of Ciprofloxacin Salicylate: SCXRD Structure Determination, Solid Characterization, Water Stability, Solubility, and Dissolution Study. *Crystals* **2020**, *10*, 349.
